# Glucose availability determines silver nanoparticles toxicity in HepG2

**DOI:** 10.1186/s12951-015-0132-2

**Published:** 2015-10-22

**Authors:** Mariusz Zuberek, Dominika Wojciechowska, Damian Krzyzanowski, Sylwia Meczynska-Wielgosz, Marcin Kruszewski, Agnieszka Grzelak

**Affiliations:** Department of Molecular Biophysics, Faculty of Biology and Environmental Protection, University of Lodz, Banacha 12/16, 90-237 Lodz, Poland; Institute of Nuclear Chemistry and Technology, Dorodna 16, 03-195 Warsaw, Poland; Faculty of Medicine, University of Information Technology and Management in Rzeszow, Sucharskiego 2, 35-225 Rzeszow, Poland; Department of Molecular Biology and Translational Research, Institute of Rural Health, Jaczewskiego 2, 20-090 Lublin, Poland

**Keywords:** Nanosilver, Oxidative stress, Antioxidant enzymes activity, Warburg effect

## Abstract

**Background:**

The increasing body of evidence suggest that nanomaterials toxicity is associated with generation of oxidative stress. In this paper we investigated the role of respiration in silver nanoparticles (AgNPs) generated oxidative stress and toxicity. Since cancer cells rely on glucose as the main source of energy supply, glucose availability might be an important determinant of NPs toxicity.

**Methods:**

AgNPs of 20 nm nominal diameter were used as a model NPs. HepG2 cells were cultured in the media with high (25 mM) or low (5.5 mM) glucose content and treated with 20 nm AgNPs. AgNPs-induced toxicity was tested by neutral red assay. Generation of H_2_O_2_ in mitochondria was evaluated by use of mitochondria specific protein indicator HyPer-Mito. Expression of a 77 oxidative stress related genes was assessed by qPCR. The activity of antioxidant enzymes was estimated colorimetrically by dedicated methods in cell homogenates.

**Results:**

AgNPs-induced dose-dependent generation of H_2_O_2_ and toxicity was observed. Toxicity of AgNPs towards cells maintained in the low glucose medium was significantly lower than the toxicity towards cells growing in the high glucose concentration. Scarceness of glucose supply resulted in upregulation of the endogenous antioxidant defence mechanisms that in turn alleviated AgNPs dependent ROS generation and toxicity.

**Conclusion:**

Glucose availability can modify toxicity of AgNPs via elevation of antioxidant defence triggered by oxidative stress resulted from enhanced oxidative phosphorylation in mitochondria and associated generation of ROS. Presented results strengthen the idea of strong linkage between NPs toxicity and intracellular respiration and possibly other mitochondria dependent processes.

**Electronic supplementary material:**

The online version of this article (doi:10.1186/s12951-015-0132-2) contains supplementary material, which is available to authorized users.

## Background

Nanoparticles (NPs) present in the environment, both of natural origin or anthropogenic, may have a significant impact on human health. Presence of the NPs in the body causes pathophysiological changes that might contribute to the development of cancer [1], cardiovascular diseases [2], respiratory tract inflammation [3], neurodegenerative diseases [4, 5], and many other pathologies [6].

Silver NPs (AgNPs) are among the one of the widest use in everyday life products, despite their reported adverse effects against various cell lines and organisms. The accumulating body of evidence suggests that harmful action of AgNPs is associated with the induction of oxidative stress [7, 8].

Oxidative stress is the most often described as an imbalance in cellular production and consumption of reactive oxygen species (ROS). Although ROS play an important role in many physiological processes, the redox imbalance is associated with many pathologies, such as Parkinson’s disease [9], Crohn’s disease, skin disease mediated by T cells, diabetes, cancer, Leigh syndrome and other mitochondrial diseases. Occurrence of the oxidative stress is often associated with disturbances in metabolic processes, such as deregulation of mitochondrial respiratory chain and/or glycolysis. Especially, concentration of glucose has a significant effect on cellular metabolism, as increased glucose level results in switching of cells metabolism from oxidative phosphorylation (OXPHOS) to glycolysis in various cell types [10], that in turn increases ROS production [11]. A proposed mechanism for this phenomenon involves an increase in intracellular calcium concentration resulting in mitochondrial fission through the function of dynamin-like protein 1 [12], that leads to apoptosis [13]. It was reported that just 2-times higher glucose concentration (50 mM) than this used in standard high-glucose DMEM leads to apoptosis of HepG2 cells [14]. Results of in vitro studies on the link between increased glucose concentration and inhibition of cell proliferation in the model cell lines correlate with in vivo results. In patients with type 2 diabetes a massive loss of beta-cells is observed, which is associated with oxidative stress induced by inhibition of glucose-6-phosphatase [15].

In physiological conditions majority of normal cells rely on OXPHOS, whereas cancer cells metabolism is based mostly on glycolysis (so-called Warburg effect) [16]. The Warburg effect may be an adaptation to the limited oxygen supply, as an early development of cancer cells usually takes place in hypoxic environment of the growing tumor that has limited blood supply until its own vasculature is developed, or be due to the shutting down of mitochondria to prevent the apoptosis [17].

Since many reports describe induction of oxidative stress by NPs and there is a number of premises linking the toxicity of NPs to oxidative stress [18, 19], with particular emphasis on ROS generation in the mitochondria, it is of great interest to ascertain, whether the availability of glucose can modify NPs toxicity. Thus the aim of this study was to determine how the glucose availability and associated changes in redox balance will affect the toxicity of NPs.

## Results

### Silver nanoparticles characteristic

Nanoparticle hydrodynamic diameter and zeta potential was measured by DLS. Hydrodynamic diameter of AgNPs of nominal size 20 nm was approximately 8 times higher when measured in albumin containing buffer. The increase of NP’s hydrodynamic diameter in protein containing medium is well recognized phenomenon usually ascribed to formation of protein corona [20]. High negative zeta potential value (−47.6 mV) confers high stability of the AgNPs dispersion and its resistance to aggregation. This was further confirmed by longitudinal analysis. Neither hydrodynamic diameter, zeta potential nor polydispersity index changed markedly over the 2 h that indicate no agglomeration (Table 1). Similar AgNPs behaviour and dispersion stability was previously observed in different cell culture media [21].

### Silver nanoparticles toxicity in high- and low-glucose culture medium

**Fig. 1:**
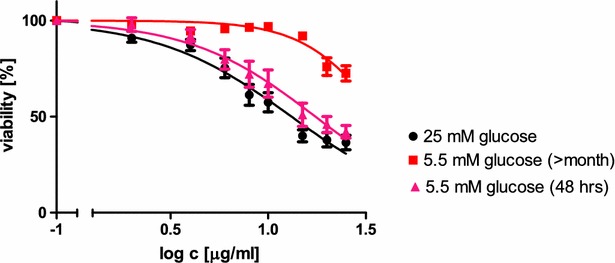
Toxicity of AgNPs (2.5–50 µg/cm^3^) for HepG2 cells cultured in the high- or low-glucose medium. Curves were compared with extra sum of square F test, p = 0.0007, N = 12 (5.5 mM glucose for 30 days, 25 mM glucose) and 9 [5.5 mM glucose (48 h)]. *Graphed points* represent means with SD

Figure 1 shows the survival of HepG2 cells cultured in high- or low-glucose DMEM for 30 days or 24 h, followed by a 24 h-treatment with AgNPs. Comparison of the survival curves reveals a statistically significant protective effect of long term culture in low glucose medium against AgNPs induced toxicity. Transferring cells to low glucose medium for 24 h before the AgNPs treatment resulted in the similar, but yet smaller, not significant effect.

**Table 1:** Hydrodynamic diameter, zeta potential and aggregation over 2 h of silver nanoparticles used in this work of nominal size 20 nm

Time (h)	Hydrodynamic diameter (nm)	Polydispersity index	Zeta potential (mV)
0	160.3 ± 2.1	0.31 ± 0.04	−47.6 ± 0.5
0.5	156.1 ± 5.3	0.31 ± 0.03	
1	160.5 ± 3.4	0.30 ± 0.01	
2	151.8 ± 4.2^a^	0.30 ± 0.03	

Results are presented as a mean ± SD. Means were compared by Student’s *t* test (n = 3)

^a^Denotes statistically important difference from the control (time 0 h)

The subsequent analysis of cell survival curves showed an decreasing difference in the effective concentrations of AgNPs necessary to induce toxicity at the particular level, between cells cultured on high- and low-glucose DMEM and cells cultured in low glucose medium for the long and short time (Table 2). The effective concentration of AgNPs, which resulted in 90 % survival of HepG2 cells cultured for 1 month in low glucose medium, was 5.5-times higher than this for cells incubated in low glucose DMEM for 24 h and 7.6-times higher than this for cells maintained in regular, high glucose DMEM. On the contrary, the predicted effective concentration of AgNPs, which resulted in 10 % survival of long term low glucose cells was only 1.4-fold higher than those for short term low glucose or long term high glucose. In preliminary experiments, we checked if the different glucose content in the culture medium affects the growth rate of HepG2 cells. No statistically important differences were found (Student’s “t” test, data not shown).

**Table 2:** The AgNP concentrations necessary to induce cell death at the given survival level

Dead cells %	AgNP concentrations necessary to induce cell death at the given survival level (µg/cm^3^)		
	High glucose culture	Low glucose culture (24 h)	Low glucose culture (30 days)
10^b^	1.81 ± 0.41	2.52 ± 0.57	13.84 ± 2.05
25^b^	4.60 ± 0.58	5.82 ± 0.73	22.91 ± 1.93
50^b^	11.70 ± 0.93	13.45 ± 1.15	37.94 ± 7.63
75^a^	29.75 ± 4.46	31.06 ± 4.99	62.83 ± 22.235
90^a^	75,61 ± 19.35	71.77 ± 19.33	104 ± 21.63

Values of each IC were compared by ANOVA accompanied by Tukey’s post test

Values represent means ± half of respective confidence intrerval, α = 0.05, n = 3

^a^Denotes statistically important difference between low glucose (30 days) and other cultures

^b^Means that viability of each culture differs from other two

### Oxidation of reporter protein with extracellular H_2_O_2_

**Fig. 2:**
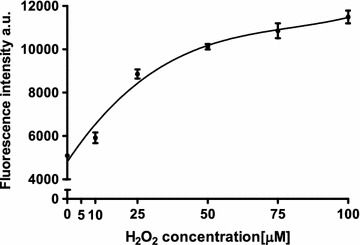
Influence of extracellular H_2_O_2_ on fluorescence intensity of reporter protein HyPer-Mito in HepG2 cells cultured on high glucose medium. *Marked points* represent mean ± SD, N = 3

In order to quantify the reactivity and sensitivity of the HyPer-mito protein in our system with respect to H_2_O_2_, we treated the HyPer-mito-transfected cells with exogenous H_2_O_2_ and measured the fluorescence of the HyPer-mito protein. We found a hyperbolic dependence of the fluorescence on the concentration of H_2_O_2_, with a saturation effect over 100 μM H_2_O_2_ (Fig. 2). Thus, the concentration of 25 µM was chosen as a positive control for the further experiments, as the one enabling the upward and downward modification of fluorescence values.

### Generation of H_2_O_2_ in mitochondria of HepG2 cells

**Fig. 3:**
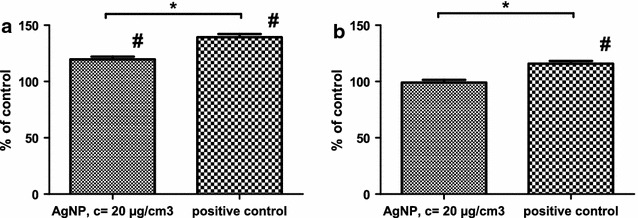
Generation of H_2_O_2_ in mitochondria measured with HyPer-mito in HepG2 cells growing on high glucose (**a**) and low glucose medium (**b**) in the presence of 25 μg/cm^3^ AgNPs. Extracellular H_2_O_2_ (25 μM) was used as a positive control. Results were normalized versus control (untreated cells) and statistical importance of differences was calculated with ANOVA accompanied by Tukey`s post hoc test. # indicates statistically important difference from the respective control, p < 0.05, N = 3. * denotes statistically important difference between AgNPs treated cell and the respective positive control, p < 0.05, N = 3. Values represent means with SD

Figure 3 presents generation of H_2_O_2_ in mitochondria of HepG2 cells cultured in different glucose concentrations and treated with AgNPs. H_2_O_2_ generation in mitochondria of cells cultured on regular, high glucose medium treated with AgNPs was higher than in control cells by 20 %, while the positive control (25 μM extracellular H_2_O_2_) was greater by 40 % (Fig. 3a). Figure 3b presents corresponding data for cells maintained on low glucose medium for 30 days. No statistically significant difference in H_2_O_2_ generation by mitochondria was observed between control cells and AgNPs-treated ones. While, the positive control showed only 16 % increase in HyPer-mito protein derived fluorescence, as compared with control cells. In control cells, without AgNPs, sustained on media with lower glucose concentration higher fluorescence intensity can be observed than in those sustained on media with high glucose content.

### Expression of oxidative stress related genes

To further examine the mechanism underlying different susceptibility of HepG2 cultured on low- and high-glucose medium to AgNPs, the expression of genes related to the cellular response to oxidative stress was evaluated (Table 3). A marked increase of expression of several genes coding proteins directly involved in oxidative defence was observed. Among those, the most notable change was observed for *ALB* (8.6-fold upregulation), *CAT* (3.5-fold upregulation), *GLRX* (2.3-fold upregulation), *GPX3* (2.2-fold upregulation), *GSTM3* (2.2-fold upregulation), *GSTM5* (72-fold upregulation), *GSTT1* (2.6-fold upregulation), *MBL2* (4.5-fold upregulation), *NCF1* (2.9-fold upregulation), and *SCARA3* (3.5-fold upregulation). Interestingly, several gene were downregulated. The most significantly down-regulated genes include: *CSDE1, GSS, OXSR1, AOX1, SRXN1, KRT1, GSTZ1, GSTA4, GPX7, SOD1, NCF2, TXNRD2, EPX, PRNP, CYGB, MPV17, PRDX3, NME5, PREX1*. Noteworthy, several of down regulated genes encode proteins involved in the glutathione synthesis and metabolism.

**Table 3:** Expression of genes involved in redox regulation in cells

Up-regulation						Down-regulation					
Gene	Expression	95 % CI	Gene	Expression	95 % CI	Gene	Expression	95 % CI	Gene	Expression	95 % CI
GSTM5	72,004	67.62–76.43	GPX3	2.199	1.88–2.48	CSDE1	0.801	0.69–0.92	NCF2	0.626	0.57–0.69
ALB	8.574	8.17–9.24	DUOX1	1.853	1.29–2.68	GSS	0.79	0.619–0.990	TXNRD2	0.564	0.48–0.67
MBL2	4.511	3.92–5.50	EPHX2	1.832	1.66–2.13	OXSR1	0.765	0.63–0.97	EPX	0.354	0.16–0.65
SCARA3	3.547	1.32–5.23	NUDT1	1.803	1.26–2.57	AOX1	0.758	0.64–0.85	PRNP	0.334	0.27–0.42
CAT	3.531	2.47–5.30	PRDX-1	1.713	1.32–2.22	SRXN1	0.734	0.56–0.89	CYGB	0.325	0.29–0.36
NCF1	2.908	1.52–5.91	PNKP	1.295	1.14–1.49	KRT1	0.704	0.53–0.98	MPV17	0.289	0.20–0.42
GSTT1	2.688	2.32–3.25	NUDT2	1.275	1.12–1.51	GSTZ1	0.702	0.53–0.92	PRDX3	0.227	0.09–0.56
GSTM3	2.389	1.94–3.03	TTN	1.254	1.12–1.43	GSTA4	0.686	0.60–0.82	NME5	0.138	0.09–0.19
SGk2	2.335	2.09–2.55	CYBA	1.24	1.17–1.32	GPX7	0.657	0.58–0.77	PREX1	0.108	0.07–0.20
GLRX	2.303	1.22–6.16	GSTM2	1.143	1.03–1.32	SOD1	0.657	0.45–0.92			

Results are presented as fold change of expression in HepG2 cells cultured on low glucose relative to expression in HepG2 cells cultured on high glucose along with 95 % confidence intervals. Only statistically important results were combined in this table (p < 0.05)

The expression of 33 genes (among 72 studied) was not affected the by long term culture on the low glucose medium. These genes include *ALOX12, ANGPTL7, APOE, ATOX1, BNIP3, DHCR24, DUSP1, EPHX2, FOXM1, GLRX2, GPR156, GPX1, GPX2, GPX4, GSR, GSTP1, LPO, MGST3, MSRA, MT2A, OXR1, PDLIM1, PRDX2, PEDX4, PRDX5, PRDX6, PTGS1, RNF7, SEPP1, SIRT2, SOD3, STK25, TXNRD1*(Additional file 1) .

### Activity of key antioxidant defence enzymes

In addition to the transcriptome analysis, enzymatic activity of the proteins playing a crucial role in the cellular antioxidant defence was evaluated (Table 4). Catalase activity was elevated 1.19 times, whereas Zn-Cu superoxide dismutase activity was elevated 1.37 times. Glutatione S-transferase and glutatione reductase activities were elevated 1.69 and 1.4 times, respectively.

**Table 4:** Enzymatic activity of the key players of cellular antioxidative defence

Enzyme	Activity (u/mg protein)	
	25 mM glucose	5.5 mM glucose
Catalase	75.19 ± 6.62	89.81 ± 7^a^
Glutathione S–transferase	0.97 ± 0.16	1.64 ± 0.26^a^
Superoxide dismutase	582 ± 75.2	803 ± 87.5^a^
Glutathione reductase	9.21 ± 1.59	12.94 ± 1.19^a^

Enzyme activities were compared with t test (n = 4, α = 0.05)

^a^Denotes statistical important difference between HepG2 cells cultured on high glucose medium and low glucose medium for 30 days. Values represent means with standard deviation

## Discussion

HepG2 cells are a well established model for study of the effects of glucose on cellular metabolism and response to different stimuli, as these cells can be easily grown in both, low- and high-glucose media [22]. Being a cell line derived from the liver, one of the target organs for nanoparticle mediated toxicity, HepG2 cells are also a well recognized model for nanotoxicity testing, including AgNPs toxicity. Toxicity of AgNPs has been demonstrated across different systems ranging from the model cell lines in vitro to small animals. In the present study, cell viability was evaluated by the Neutral red assay. This assay is less prone to artefacts than the commonly used MTT assay, as the reduction of tetrazolium dyes is strongly dependent on the metabolic state of the cell, in particular on the activity of NAD(P)H-dependent oxidoreductases [15]. Neutral red viability test is based on dye accumulation in lysosomes of viable cells and seems to be less dependent on fluctuations in the redox state of the cells. However, despite the test used, published results repeatedly confirm toxicity of AgNPs [8]. Also the current study confirmed the toxicity of AgNPs to HepG2 cells in vitro.

Analyzing the survival curves for HepG2 cells cultivated in media with different glucose concentration and treated with AgNPs we observed an increase in resistance to the NPs in cells cultured on low glucose for 30 days, as compared to cells cultured on high glucose medium or on low glucose medium for 24 h (Fig. 1). This effect is especially visible at the high survival levels, e.g. the AgNPs concentrations necessary to reduce the survival of HepG2 cells growing on low glucose medium for 30 days to 90 and 75 % were approximately 7.6 and 5 times higher than those necessary to reduce the survival of the cells growing on high glucose medium to the same survival level, respectively (Table 2). Interestingly, the difference decreases along with the decrease of the survival level, as the estimated AgNPs concentrations necessary to reduce the survival of HepG2 cells growing on low glucose medium for 30 days to 25 and 10 % were only 2.1 and 1.4 times higher than those necessary to reduce the survival of cells growing on high glucose medium to the same survival level, respectively (Table 2). There was no difference between cells growing on low glucose medium for 24 h and those growing on high glucose medium. These results clearly point to the existence the adaptive mechanisms in low-glucose-growing cells that diminish the toxic effect of AgNPs, but also indicate a limited capacity of the adaptive mechanisms, as the difference between the low- and high-glucose growing cells decreased with the decrease of the survival level.

Although the mechanisms of the AgNPs toxicity is still under debate, it is most likely associated with the induction of oxidative stress. Induction of the oxidative stress by AgNPs has been proven in different systems in vitro and in vivo [7, 8, 22]. It is usually associated with excessive generation of free radicals due to the dysfunction of mitochondria [23] and/or activation of ROS generating enzymes, e.g. NADPH reductase [24]. Whereas fluorescent probes commonly used to assess ROS generation are either non-specific or difficult to target to the particular cell compartment, in this work we directly and specifically confirmed generation of H_2_O_2_ in mitochondria of AgNPs treated HepG2 cells, by the use of mitochondria targeted, H_2_O_2_ specific HyPer-mito reporter protein. AgNPs treatment of HepG2 cells growing on the standard high glucose medium resulted in the increase of H_2_O_2_ generation in the mitochondria by 40 %, as compared with control, untreated cells (Fig. 3a). This is consistent with the previous report on AgNP dependent ROS generation in HepG2 cells [21], and the report of generation of H_2_O_2_ in bronchial epithelial cells (BEAS-2B) [25]. In contrast, AgNPs treatment did not increase generation of H_2_O_2_ in HepG2 cells grown on low-glucose medium, in concordance with the survival data described above. A plausible, coming to the mind explanation of this phenomenon is an intensification of the antioxidant defence systems that diminish AgNPs-induced ROS toxicity. In parallel with this interpretation, treatment with exogenous H_2_O_2_ resulted in an only 16 % increase in H_2_O_2_ detected inside mitochondria of cells grown on low glucose, whereas the same treatment resulted in 40 % increase of H_2_O_2_ detectable in mitochondria of the high glucose growing cells.

Indeed, analysis of the enzymatic activity of the key enzymes of the cellular antioxidant defence system revealed statistically significant increase of the activities of all tested enzymes, including catalase, glutathione S-transferase, glutathione reductase and superoxide dismutase (Table 4). While the acute oxidative stress usually results in a cell membrane injury and damage to macromolecules and organelles leading to cell death, the activation of antioxidant adaptive response at a moderate level of oxidative stress is well recognized phenomenon. This diverse cellular response is connected with activation of different cellular signalling pathways, such as MAP kinases cascade and/or pathways associated with redox-sensitive transcription factors, such as HIF-1, NF-κB and Nrf-2 [26–28] The activation of NF-κB transcription factor and an increased expression of its target genes was also reported in HepG2 cells treated with AgNPs [29]. In addition, adaptation of HepG2 cells to AgNPs induced oxidative stress has been already proposed as a mechanism explaining the differences in response to nanosilver between HepG2 and A549 cell lines [30]. In that work a different AgNPs susceptibility of HepG2 and A549 cells was linked to upregulation of pro-proliferative and anti-apoptotic signalling pathways.

In our experimental setup, an observed increase of antioxidant enzymes activities resulted as an adaptation to low glucose condition and accompanied increase of oxidative stress. The impact of glucose on cell metabolism has been widely studied and there are numerous data on the effects of glucose availability on oxidative stress induction. High glucose concentration leads to increased protein glycation, but provides more sustainable environment for cell growth. However, increased mitochondrial protein glycation and accumulation of advanced glycation end-products may lead to mitochondrial dysfunction and oxidative stress, as has been shown on *C. elegans* [31] and human cells [32].

On the other hand, ROS generation has been also associated with low glucose availability [33], which is consistent with reports of switching cell metabolism from glycolysis to OXPHOS [10, 34]. In physiological conditions the prevalent way for energy supply in cancer cells is glycolysis, thus glucose scarcity forces the metabolic switch back to OXPHOS (so-called the Warburg effect) [35]. The phenomenon of metabolic switch between glycolysis and respiratory chain in response to glucose availability was observed in a variety of experimental setups, ranging from yeast to mammalian cell lines [36, 37]. The Warburg effect was firstly explained by irreversible damage to the elements of oxygen-dependent pathway of OXPHOS in cancer cells. However, this explanation was questioned by the recent investigations showing an intact functionality of mitochondrial OXPHOS in many cancer cells [38–40] and the studies describing similar effect in non-cancer, proliferating cells, which were not supposed to have the OXPHOS pathway irreversibly damaged [41]. Moreover, many authors consider the Warburg effect as a result of suppression of mitochondrial OXPHOS due to enhanced glycolysis rather than defects in its functionality. If glycolysis is inhibited in cancer cells, the function of mitochondrial OXPHOS can be restored [38, 42, 43]. Indeed, also in our experimental setup, depletion of glucose supply resulted in metabolic switch and enhanced production of H_2_O_2_ in mitochondria due to the OXPHOS. As oxidative stress prolonged, cells adopted to the new situation by elevating the activity of key antioxidant defence enzymes (Table 4).

This was further confirmed by the transcriptome analysis. The level of catalase gene mRNA (*CAT*) was elevated 3.5-times in cells cultured in the low glucose medium, as compared to cells cultured in high glucose medium. Catalase is a key peroxisome antioxidant enzyme detoxifying H_2_O_2_ and plays a protective role against H_2_O_2_-induced oxidation in nearly all aerobic organisms. A similar increase of the *CAT* expression was observed in response to oxidative stress [44] and low glucose condition [45]. Glucose scarcity resulted also in upregulation of the Scavenger Receptor Class A, Member 3 (*SCARA3*) mRNA level (3.5 times). Being a macrophage scavenger receptor-like protein, the Scara3 protein has been shown to deplete ROS and other harmful products of oxidation, thus playing an important role in protecting cells from oxidative stress. Brown et al. [46] reported an increase of SCARA3 expression in cells treated with H_2_O_2_.

Of note is the 50-times increase of mRNA level of *GSTM5* gene that belongs to the mu class of glutathione *S*-transferases (Table 3), although accompanied only by 1.7 times higher activity of the enzyme (Table 4). The Gstm5 protein functions in detoxification of electrophilic compounds, including carcinogens, therapeutic drugs, environmental toxins and products of oxidative stress, by conjugation with reduced glutathione. Overexpression of *GSTM5* has been already reported due to the generation of ROS in low-glucose medium [47]. A similar phenomenon was found in AgNPs treated *Arabidopsis thaliana* roots [48] that suggest a common mechanisms of response to the increased mitochondrial generation of ROS. A marked up-regulation of expression was also observed for albumin gene (*ALB*). Albumin is a liver-derived, the most abundant plasma protein well recognized for its antioxidant properties [49, 50], Human serum albumin was shown to prevent neuronal death in murine cortical cell cultures exposed to oxidative stress generated by H_2_O_2_ [51].

In addition to antioxidant proteins and enzymes, another markedly up-regulated gene was a Mannose-Binding Lectin (Protein C) 2, Soluble (*MBL2)*, which plays an important role in innate immune defence system. Another important function of MBL2 protein is binding to the apoptotic and necrotic cells that facilitate their uptake by macrophages. MBL2 is produced in the liver in a response to infection, typically associated with generation of oxidative stress [52].

Noteworthy, although Zn-Cu superoxide dismutase activity was still elevated, transcritpome analysis revealed that its mRNA transcription was already shut down (Tables 3, 4). This discrepancy can be easily explained by increased activity of mitochondrial respiratory chain induced by low glucose condition and mutually associated increase of its unwanted by-product, superoxide anion radical. Moreover, the Neutrophil Cytosol Factor 1 (*NCF1*) mRNA level was increased 2.9 times. NCF1 protein is a subunit of NADPH oxidase, the enzyme also producing superoxide anion radical. Superoxide anion radical is subsequently converted into H_2_O_2_ by superoxide dismutase. As superoxide anion radical is less reactive than H_2_O_2_-derived hydroxyl radical and due to the negative charge does not easily cross the cell membranes, it is probably less dangerous and more favourable as an excess, resident ROS than H_2_O_2_. In agreement, catalase activity and transcription was elevated (Tables 3, 4).

Surprisingly, low glucose concentration in culture medium resulted in down-regulation of several genes involved in synthesis and metabolism of glutathione (*GSS, GSTZ1, GSTA4, GPX*), This suggest that general detoxification of ROS generated in low glucose condition is carried out with fast enzymatic reactions rather than small molecule antioxidants, such as glutathione. Down-regulation of glutathione synthesis is in line with up-regulation of glutathione S-transferase, that deplete the reduced glutathione by conjugating with nucleophiles, the conjugates are further removed by ABC-type transporters [53]. Since glutathione may act as an anti- and pro-oxidant [54], it seems favourable to diminish glutathione redox-cycling mechanism in chronic oxidative stress condition. Moreover, in chronic oxidative stress GSH depletion may promote pro-survival pathways. Whereas, a rapid GSH depletion induces pro-apoptotic pathway [55], its slow and prolonged depletion triggers NFkB signalling pathway and associated anti-oxidative and pro-survival response [56]. Activation of NFkB signalling pathway is repeatedly reported in oxidative stress condition of different origin, including AgNPs treatment [29]. However, it must be remembered that different signalling pathways often stimulated by the same condition (e.g. oxidative stress) may interact each other, as recently reviewed for NFkB and NRF/KEAP signalling pathways [57] and many others [58] or even counteract, e.g. mitigation of oxidative stress induced NFkB pathway activity by HIF-1 [59]. Thus, a net effect of signalling pathways stimulation is a result of pathways crosstalk and local, intracellular context.

## Conclusions

We have shown that glucose availability can modify toxicity of AgNPs via elevation of antioxidant defence triggered by oxidative stress resulted from enhanced OXPHOS in mitochonddria and associated generation of ROS. Since the mechanism does not depend on the toxic factor but the activity of mitochondria, it seems to be universal, regarding not only AgNPs, but all oxidative stress generating agents, e.g. another nanomaterials, pesticides and many others. Moreover, our results suggest that cells relying strongly on glycolysis (such as cancer cells) might be more prone to toxic action of nanomaterials that normal cells, whose metabolism is based on the OXPHOS. Further, higher survival of cells cultured in environment forcing the activation of mitochondrial respiratory chain, as compared with glycolysis based ones, gives a good prognosis for use of nanomaterials in medical and pharmaceutical application.

On the other hand, few crucial cell types in the human body strongly rely on glycolysis, including brain, muscle and liver cells [60] that makes them more susceptible to the toxic action of nanomaterials than OXPHOS based cells, unless have other mechanisms elevating antioxidant response.

Finally, our results suggest that local microenvironment, such as local availability of glucose, might be a factor promoting an adaptive abilities of cells, and modifying their response to xenobiotics, including nanomaterials.

## Methods

### Cell culture and treatment

HepG2 cell line was purchased from the American Type Culture Collection (ATCC). The cells were cultured in 75 cm^3^ Nunclon flasks in glucose supplemented Dulbecco’s Modified Eagle Medium (DMEM). Medium described in this paper as a high-glucose medium contained glucose at the concentration of 4.5 g/dm^3^ (25 mM), whereas low glucose medium contained 1 g/dm^3^ glucose (5.5 mM). Both variants of the medium were supplemented with 10 % FCS (Gibco). Glucose concentrations roughly reflect average blood sugar level and heavy hyperglycaemic level [61]. Cells were maintained in a 5 % CO_2_ atmosphere at 37 °C at 95 % relative humidity. Cells described further as being cultured for long period of time on low glucose were passaged for 30 days before experiments in medium with 5.5 mM glucose. Cells referred to as sustained for short period of time on low glucose were incubated in medium with 5.5 mM glucose for 48 h, including AgNPs treatment.

### Silver nanoparticle preparation

The stock suspension (2 mg/cm^3^) of the AgNPs of a 20 nm nominal diameter (PlasmaChem, Germany) was prepared as previously described [13]. In brief, AgNPs were triturated in agate mortar, then 2 mg of NPs was suspended in 800 µL of water. Suspension was sonicated (4.2 kJ/cm^3^) with OmniRuptor 4000 and then 100 µL of 15 % albumin which was followed by addition of 100 µL of tenfold concentrated PBS (1.37 M NaCl, 27 mM KCl, 80 mM Na_2_HPO_4_, 20 mM KH_2_PO_4_). Intermediate dilutions were made in DMEM with the corresponding high or low glucose content.

Nanoparticles’ hydrodynamic diameter and zeta potential was measured by dynamic light scattering (DLS, Malvern, UK). Both the hydrodynamic diameter and zeta potential of the samples were measured at 25 °C with a scattering angle of 173°. The particle concentration for hydrodynamic diameter measurements in each sample was 2 mg/cm^3^ in the preparation buffer. Measurements were done in triplicate with over 14 sub-runs. The pH value of the suspensions was 7.1.

A detailed characterization of this batch of AgNPs, including different preparation protocols, dispersion, aggregation and stability in different culture media, as well as silver ion release, was previously described in [21, 62]

### Measurement of H_2_O_2_ generation in mitochondria

HepG2 cells were suspended at a density of 1 × 10^6^ cells/cm^3^ and centrifuged for 10 min at 200×*g* in 1.5 cm^3^ Eppendorf tube. The supernatant was removed, the cells were resuspended in 0.8 cm^3^ of hypoosmolar buffer (Eppendorf cat no 940002001), and 10 μg of HyPer-mito plasmid (EVROGEN, Russia, cat.# FP942) was added. Cells were transferred to a cuvette and electroporated with three 10 μs pulses with a voltage of 500 V (Multiporator^®^, Eppendorf).

After electroporation cells were plated on 6-well plate (Nunclon) in 3 cm^3^ of DMEM medium with an appropriate glucose concentration (5.5 or 25 mM). After 24 h of culture cells were trypsinized with 0.25 cm^3^ of 0.25 % trypsin, suspended in 0.75 cm^3^ of appropriate DMEM medium and transferred to cytometry tubes. Fluorescence intensity of transfected cells was measured using a LSRII flow cytometer in the FITC channel. Transfection efficiency was found to be at the level of 60–70 %. For further analysis only cells that exhibited fluorescence above the maximum autofluorescence of mock transfected cells were used.

To check the approximate range of H_2_O_2_ detection by the product of the reporter vector, HyPer-mito plasmid transfected HepG2 cells were cultured in 25 mM glucose and titrated with extracellular H_2_O_2_. Five minutes after H_2_O_2_ addition cells’ fluorescence was read in a LSRII cytometer.

### Neutral red viability test

Cells were plated on 96-well plates (Nunclon) at a density of 15 000 cells per well in a final volume of 0.1 cm^3^. After 24 h, an appropriate aliquot of AgNPs was added to achieve concentrations in range from 2.5 to 50 µg/cm^3^ in a final volume of 0.2 cm^3^ After next 24 h incubation, the medium was removed and cells were washed twice with 0.15 cm^3^ per well of PBS solution. The cells were then flooded with 0.1 cm^3^ of neutral red solution (50 µg/cm^3^ neutral red in the culture medium). After a 4-h incubation at 37 °C in an atmosphere of 5 % CO_2_ neutral red solution was discarded, the cells washed twice with 0.15 cm^3^ PBS and 0.15 cm^3^ of fixative (50 % of 96 % ethanol, 49 % H_2_O, 1 % acetic acid) was added to each well. The plate was shaken for 15 min and the absorbance was measured at a wavelength of 540 nm using an EnVision^®^ Multilabel Reader (Perkin-Elmer).

### Gene expression analysis

Total RNA was isolated from 10^6^ HepG2 cells employing MagNA Pure LC 2.0 Instrument (Roche) according to the manufacturer’s protocol. Genomic DNA was removed by DNase I digestion (RNase free DNAse, Life Technologies) and 1 µg of the total RNA was reverse-transcribed using the SuperScript™ III First-Strand Synthesis SuperMix (Life Technologies). qPCR analysis was performed with C1000 Thermal Cycler–CFX96 Real-Time System (BioRad).

The human oxidative stress library (HOSL-1) primers (RealTimePrimers.com) were used to assess the expression of oxidative stress related genes using iQ SYBR Green Supermix (BioRad). After 3 min of an initial activation and denaturation step at 95 °C, 40 cycles of denaturation at 95 °C for 10 s, annealing/extension at 60 °C for 45 s followed by melt curve analysis (55–95 °C, 0.5 °C increment, 5 s/step) were performed. cDNA was omitted in non-template control. The mRNA level was calculated using the ΔΔct method (Rest, Qiagen), and normalized to the housekeeping gene (*ACTB),* as a control.

### Assessment of antioxidant enzymes activity in cells homogenate

For biochemical analysis cells were plated in 75 cm^3^ Nunc flasks, 3 × 10^6^ cells per flask and cultured in DMEM with low or high glucose concentration at 5 % CO_2_ atmosphere, 37 °C and 95 % relative humidity. For enzymatic activity estimation, the cells were harvested by trypsinization when cultures reached 80 % confluence (roughly after 48 h culture), lysed with 0.1 % Triton/1 mM EDTA solution and frozen overnight at −20 °C. All measurements were conducted in 96-well NUNC plates in an EON spectrophotometer (BioTek).

Catalase activity of samples was determined by assessing their ability to decompose H_2_O_2_ as previously described [63]. Gluthatione S-transferase activity was determined with colorimetric method as described by Rise-Evans et al. [64]. SOD activity was assessed by the NBT reduction method [65]. Glutathione reductase was assayed on the basis of NADPH oxidation [66]. The results were referenced to protein concentration in the samples and enzymatic activity was calculated by comparison in comprehension with enzyme standards. All methods were adapted to 96-well microplates by scaling down the volume of reagents.

### Statistical evaluation

All statistical evaluations were made using a GraphPad Prism 5, Microsoft Excel and REST software. Curves graphed in Fig. 1 were compared utilizing extra sum of square F test. Difference between various inhibitory concentrations summarized in Table 2 were calculated by ANOVA with Tukey`s post hoc test. Statistical significance of differences in generation of hydrogen peroxide induced by different treatments, summarized in Fig. 3 was calculated with ANOVA followed by Tukey`s post hoc test. The expression ratio results (Table 3) of the investigated transcripts were tested for significance by a Pair Wise Fixed Reallocation Randomisation Test and error is expressed as a standard error (SE) estimated via Taylor algorithm (REST software) [67]. Enzymes activities summarised in Table 4 were compared with t test.

## Additional file

10.1186/s12951-015-0132-2 Expression on mRNA level of all tested genes. Results are shown as fold change of expression in cells sustained on low glucose medium (5.5 mM) when compared with cells sustained on high glucose medium (25 mM).

## Abbreviations

AgNPs: silver nanoparticles
DLS: dynamic light scattering
OXPHOS: oxidative phosphorylation
ROS: reactive oxygen species
HIF-1: hypoxia inducible factor 1
NF-κB: nuclear factor kappa B
Nrf-2: nuclear factor (erythroid-derived 2)-like 2 (NFE2L2)
