# Supplementary material for: Glucose availability determines silver nanoparticles toxicity in HepG2
Source: J Nanobiotechnology. 2015 Oct 22;13:72. doi: 10.1186/s12951-015-0132-2 (PMC4618757; doi:10.1186/s12951-015-0132-2)
Supplement: Supplementary file 1 — 10.1186/s12951-015-0132-2 Expression on mRNA level of all tested genes. Results are shown as fold change of expression in cells sustained on low glucose medium (5.5 mM) when compared with cells sustained on high glucose medium (25 mM). [file 12951_2015_132_MOESM1_ESM.docx]

| Gene | | Expression | Std. Error | Result | |
| --- | --- | --- | --- | --- | --- |
| ALB | Albumin | 8,574 | 8,184 - 9,190 | UP | |
| ALOX12 | Arachidonate 12-lipoxygenase | 1,498 | 1,367 - 1,612 |  | |
| ANGPTL7 | Angiopoietin-like 7 | 1,197 | 1,171 - 1,230 |  | |
| AOX1 | Aldehyde oxidase 1 | 0,758 | 0,654 - 0,833 | DOWN | |
| APOE | Apolipoprotein E | 1,159 | 1,092 - 1,212 |  | |
| ATOX1 | Antioxidant protein 1 homolog (yeast) | 2,014 | 1,920 - 2,138 |  | |
| BNIP3 | BNIP3 BCL2/adenovirus E1B 19kDa interacting protein 3 | 1,2 | 1,024 - 1,360 |  | |
| CAT | Catalase | 3,531 | 2,777 - 4,364 | UP | |
| CSDE1 | Cold shock domain containing E1, RNA-binding | 0,801 | 0,720 - 0,879 | DOWN | |
| CYBA | Cytochrome b-245, alpha polypeptide | 1,24 | 1,200 - 1,296 | UP | |
| CYGB | Cytoglobin | 0,325 | 0,307 - 0,351 | DOWN | |
| DHCR24 | 24-dehydrocholesterol reductase | 2,194 | 1,990 - 2,406 |  | |
| DUOX1 | Dual oxidase 1 | 1,853 | 1,496 - 2,331 | UP | |
| DUSP1 | Dual oxidase 2 | 0,786 | 0,675 - 0,894 |  | |
| EPHX2 | Epoxide hydrolase 2, cytoplasmic | 1,832 | 1,682 - 1,952 |  | |
| EPX | Eosinophil peroxidase | 0,354 | 0,188 - 0,579 | DOWN | |
| FOXM1 | Forkhead box M1 | 1,485 | 1,318 - 1,636 |  | |
| GLRX | Glutaredoxin | 2,303 | 1,359 - 5,392 | UP | |
| GLRX2 | Glutaredoxin 2 | 1,566 | 0,713 - 4,299 |  | |
| GPR156 | G protein-coupled receptor 156 | 0,537 | 0,388 - 0,791 |  | |
| GPX1 | Glutathione peroxidase 1 | 0,654 | 0,541 - 0,871 |  | |
| GPX2 | Glutathione peroxidase 2 | 2,335 | 2,130 - 2,526 |  | |
| GPX3 | Glutathione peroxidase 3 | 2,199 | 1,952 - 2,406 | UP | |
| GPX4 | Glutathione peroxidase 4 | 1,225 | 1,101 - 1,427 |  | |
| GPX7 | Glutathione peroxidase 7 | 0,657 | 0,596 - 0,735 | DOWN | |
| GSR | Glutathione reductase | 0,794 | 0,637 - 0,949 |  | |
| GSS | Glutathione synthetase | 0,79 | 0,667 - 0,956 | DOWN | |
| GSTA4 | Glutathione S-transferase A4 | 0,686 | 0,617 - 0,754 | DOWN | |
| GSTM2 | Glutathione S-transferase M2 (muscle) | 1,143 | 1,048 - 1,227 | UP | |
| GSTM3 | Glutathione S-transferase M3 (brain) | 2,389 | 2,064 - 2,835 | UP | |
| GSTM5 | Glutathione S-transferase mu 5 | 72,004 | 67,620 - 76,430 | | UP |
| GSTP1 | Glutathione S-transferase pi | 0,761 | 0,337 - 1,478 |  | |
| GSTT1 | Glutathione S-transferase theta 1 | 2,688 | 2,391 - 3,062 | UP | |
| GSTZ1 | Glutathione transferase zeta 1 | 0,702 | 0,573 - 0,851 | DOWN | |
| KRT1 | Keratin 1 (epidermolytic hyperkeratosis) | 0,704 | 0,580 - 0,913 | DOWN | |
| LPO | Lactoperoxidase | 0,052 | 0,038 - 0,081 |  | |
| MBL2 | Mannose-binding lectin (protein C) 2, soluble | 4,511 | 3,990 - 5,305 | UP | |
| MGST3 | Microsomal glutathione S-transferase 3 | 0,557 | 0,343 - 0,827 |  | |
| MPV17 | MpV17 mitochondrial inner membrane protein | 0,289 | 0,211 - 0,370 | DOWN | |
| MSRA | Methionine sulfoxide reductase A | 0,897 | 0,728 - 1,132 |  | |
| MT2A | Metallothionein 2A | 0,471 | 0,338 - 0,756 |  | |
| NCF1 | Neutrophil cytosolic factor 1 | 2,908 | 1,707 - 4,869 | UP | |
| NCF2 | Neutrophil cytosolic factor 2 | 0,626 | 0,586 - 0,660 | DOWN | |
| NME5 | Non-metastatic cells 5 | 0,138 | 0,091 - 0,178 | DOWN | |
| NUDT1 | Nudix -type motif 1 | 1,803 | 1,419 - 2,367 | UP | |
| NUDT2 | Nudix -type motif 2 | 1,275 | 1,152 - 1,403 | UP | |
| OXR1 | Oxidation resistance 1 | 1,13 | 1,037 - 1,276 |  | |
| OXSR1 | Oxidative-stress responsive 1 | 0,765 | 0,654 - 0,873 | DOWN | |
| PDLIM1 | PDZ and LIM domain 1 (elfin) | 1,009 | 0,883 - 1,298 |  | |
| PNKP | Polynucleotide kinase 3'-phosphatase | 1,295 | 1,155 - 1,461 | UP | |
| PRDX-1 | Peroxiredoxin 1 | 1,713 | 1,450 - 2,058 | UP | |
| PRDX2 | Peroxiredoxin 2 | 0,968 | 0,720 - 1,284 |  | |
| PRDX3 | Peroxiredoxin 3 | 0,227 | 0,111 - 0,475 | DOWN | |
| PEDX4 | Peroxiredoxin 4 | 1,03 | 0,706 - 1,379 |  | |
| PRDX5 | Peroxiredoxin 5 | 0,715 | 0,445 - 1,154 |  | |
| PRDX6 | Peroxiredoxin 6 | 0,256 | 0,171 - 0,475 |  | |
| PREX1 | Phosphatidylinositol 3,4,5-trisphosphate-dependent RAC exchanger 1 | 0,108 | 0,075 - 0,146 | DOWN | |
| PRNP | Prion protein (p27-30) | 0,334 | 0,291 - 0,388 | DOWN | |
| PTGS1 | Prostaglandin-endoperoxide synthase 1 | 0,816 | 0,370 - 2,192 |  | |
| RNF7 | Ring finger protein 7 | 1,055 | 0,916 - 1,261 |  | |
| SCARA3 | Scavenger receptor class A, member 3 | 3,547 | 1,549 - 6,996 | UP | |
| SEPP1 | Selenoprotein P, plasma, 1 | 2,329 | 2,160 - 2,500 |  | |
| SGk2 | Serum/glucocorticoid regulated kinase 2 | 2,335 | 2,228 - 2,508 | UP | |
| SIRT2 | Sirtuin 2 | 1,254 | 0,848 - 2,275 |  | |
| SOD1 | Superoxide dismutase 1, soluble | 0,657 | 0,528 - 0,845 | DOWN | |
| SOD3 | SOD3 Superoxide dismutase 3, extracellular | 1,326 | 0,842 - 2,199 |  | |
| SRXN1 | Sulfiredoxin 1 homolog (S. cerevisiae) | 0,734 | 0,656 - 0,855 | DOWN | |
| STK25 | Serine/threonine kinase 25 (STE20 homolog, yeast) | 0,937 | 0,699 - 1,264 |  | |
| TTN | Titin | 1,254 | 1,149 - 1,384 | UP | |
| TXNRD1 | Thioredoxin reductase 1 | 0,434 | 0,414 - 0,458 |  | |
| TXNRD2 | Thioredoxin reductase 2 | 0,564 | 0,508 - 0,640 | DOWN | |
